# Supplementary figures and images for: Staphylococcal toxin PVL ruptures model membranes under acidic conditions through interactions with cardiolipin and phosphatidic acid
Source: PLoS Biol. 2025 Apr 15;23(4):e3003080. doi: 10.1371/journal.pbio.3003080 (PMC12052211; doi:10.1371/journal.pbio.3003080)

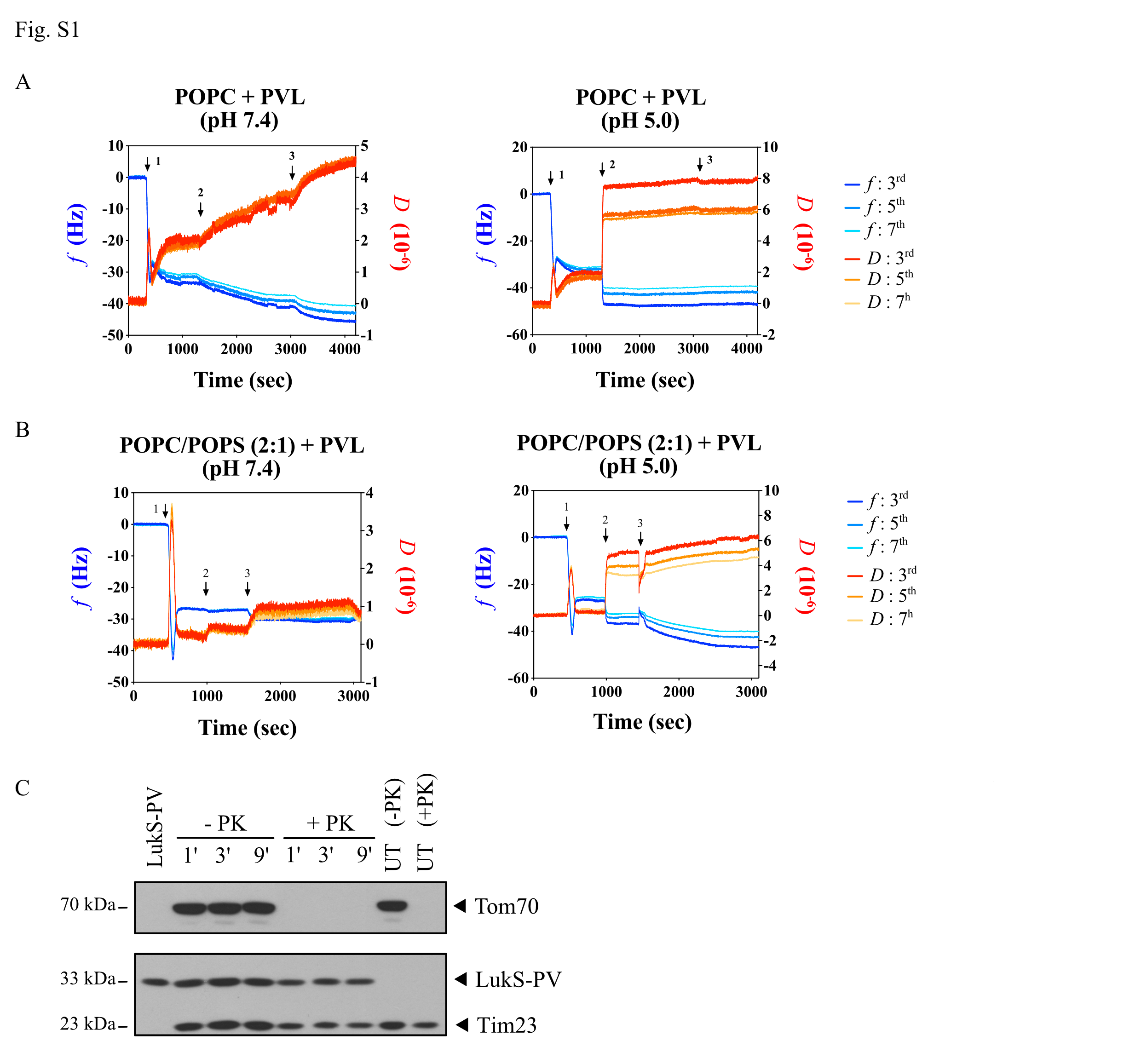

Supplement: S1 Fig — QCM-D profiles for purified PVL in different pH buffers on supported lipid bilayer made of (A) POPC or (B) POPC/POPS (w/w 2:1). Liposome addition indicated with 1↓, buffer exchange with 2↓ and PVL treatment (100 µg/ml) with 3↓. Blue: frequency; red: dissipation. Data representative of two independent experiments. (C) Mitochondria isolated from S. cerevisiae were treated with/without proteinase K after import of LukS-PV for indicated time. Mitochondrial extracts were probed with antibodies against LukS-PV, Tom70, and Tim23. The data underlying this Figure can be found in S1 Data. (TIF) [file pbio.3003080.s001.tif]

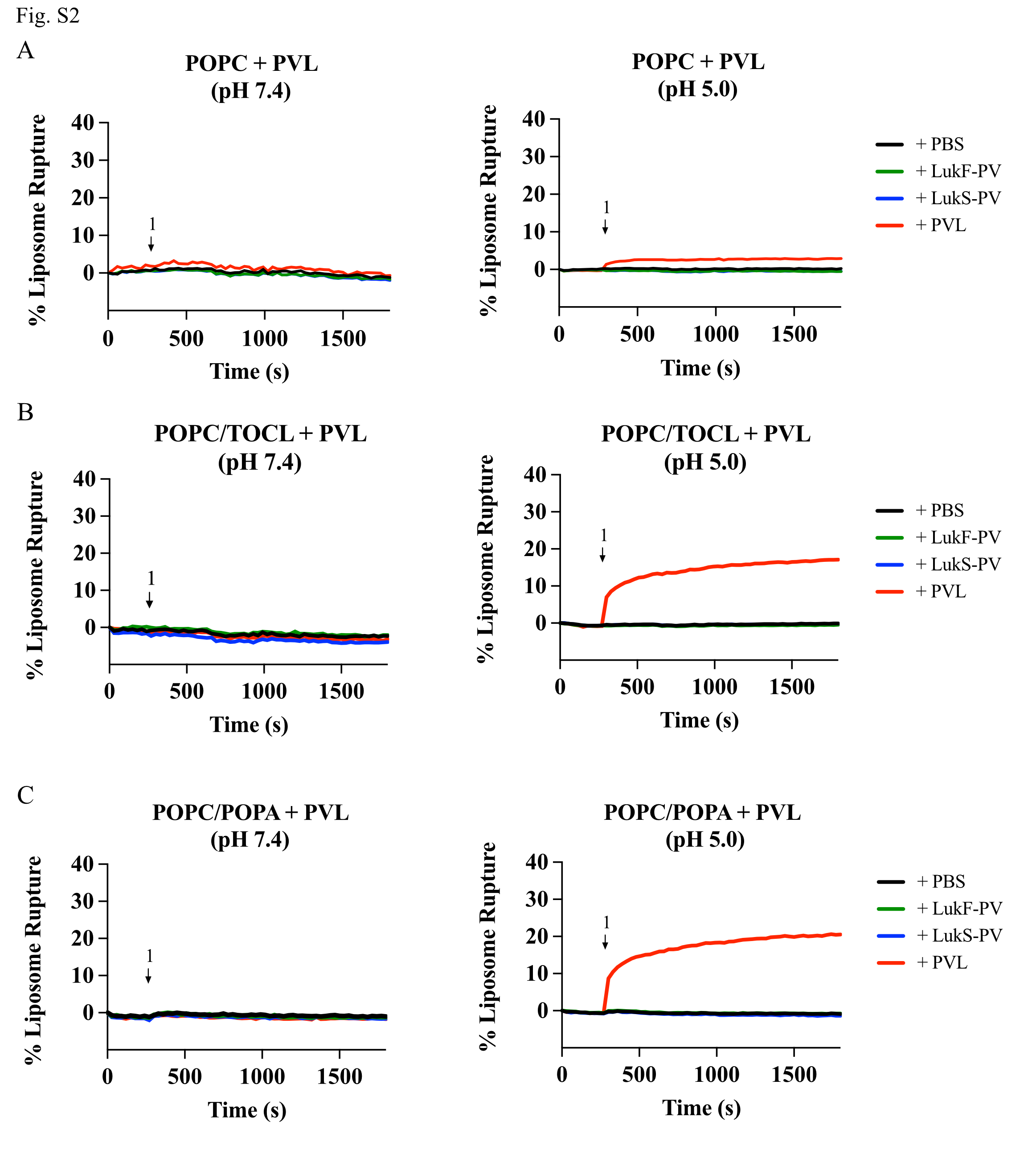

Supplement: S2 Fig — (A–B) Sulforhodamine B encapsulated liposomes of (A) POPC, (B) POPC/TOCL (w/w 2:1) or (C) POPC/POPA (w/w 3:1) were treated with purified PVL and its subunits LukS-PV and LukF-PV (1 µg/ml) in neutral or acidic buffers overtime. Increased sulforhodamine B fluorescence due to its release was determined overtime, compared to Triton X-100 treatment. PVL and subunits were added at the indicated time point (1↓). Mean from three independent experiments are shown. The data underlying this Figure can be found in S1 Data. (TIFF) [file pbio.3003080.s002.tiff]

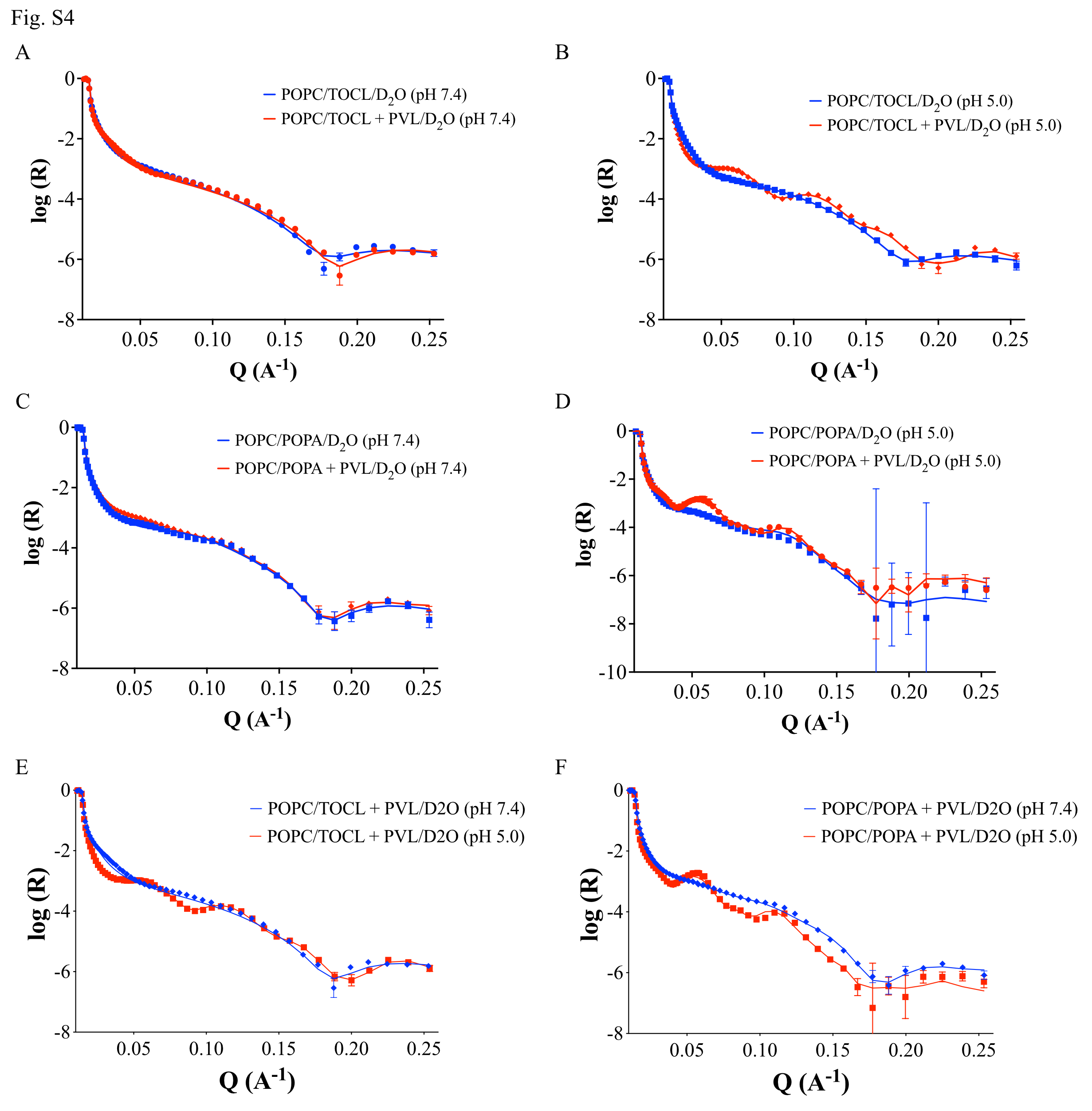

Supplement: S4 Fig — (A–D) Neutron reflectometry (NR) analysis of lipid bilayers with or without purified PVL (100 µg/ml). (A–B) POPC/TOCL (w/w 2:1) membrane in D2O buffer at (A) pH 7.4 or (B) pH 5.0 with (red curve) or without PVL (blue curve). (C–D) POPC/POPA (w/w 2:1) membrane in D2O buffer at (C) pH 7.4 or (D) pH 5.0 with (red curve) or without PVL (blue curve). (E–F) NR profiles for purified PVL interacting with (E) POPC/TOCL (w/w 2:1) and (F) POPC/POPA (w/w 2:1) membrane in D2O buffer at pH 5.0 (red curve) or 7.4 (blue curve). The data underlying this Figure can be found in S1 Data. (TIFF) [file pbio.3003080.s004.tiff]

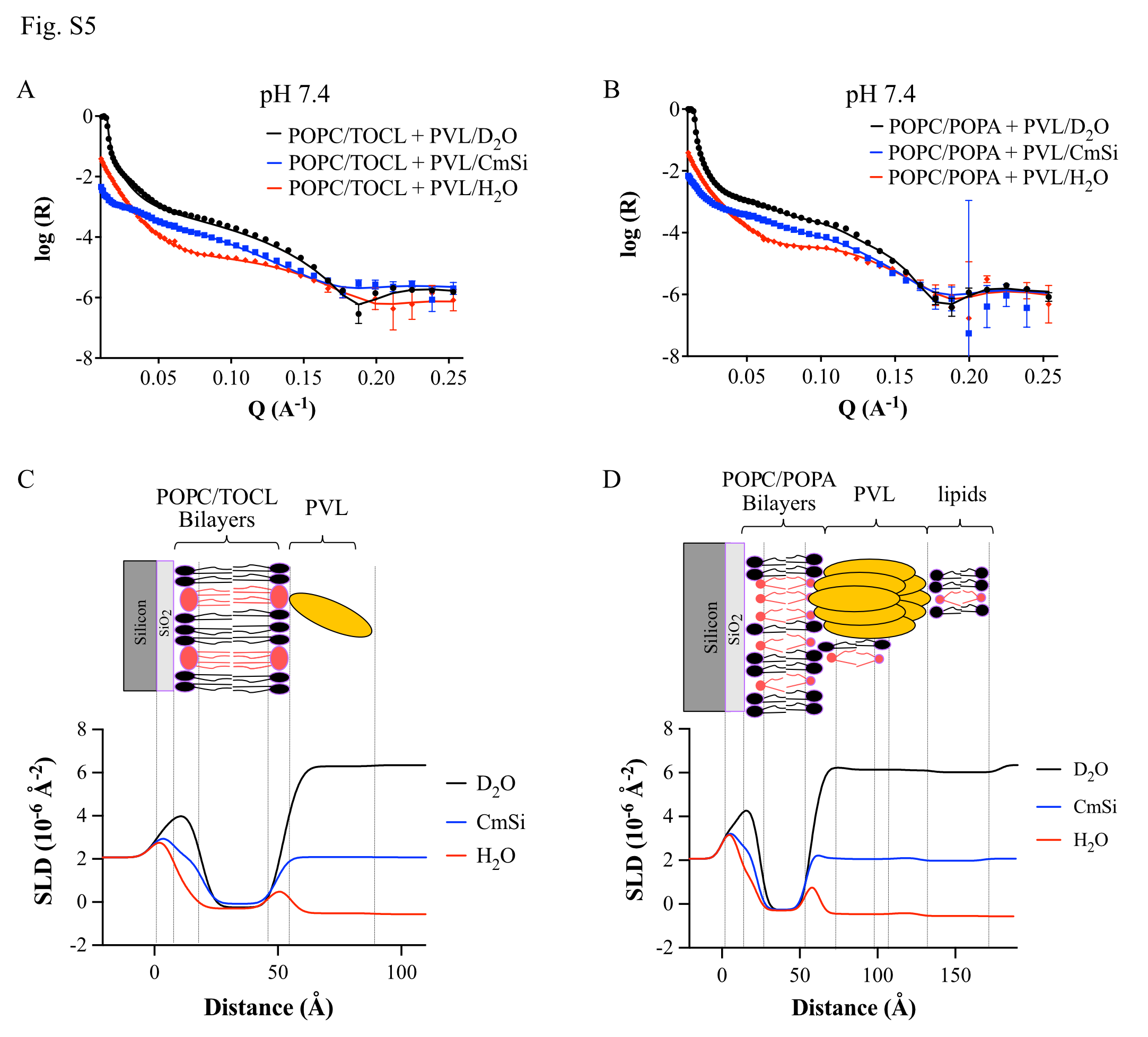

Supplement: S5 Fig — (A–B) Neutron reflectometry (NR) analysis of purified PVL (100 µg/ml) interacting with (A) POPC/TOCL (w/w 2:1) and (B) POPC/POPA (w/w 2:1) membrane in pH 7.4 buffer. Black curve: measurement in D2O buffer; Red curve: measurement in contrast matched Si buffer (CmSi); Blue curve: measurement in H2O buffer. (C–D) Scattering length density profiles with cartoons for PVL with (C) POPC/TOCL and (D) POPC/POPA membrane. Black line: measurement in D2O buffer; Red line: measurement in contrast matched Si buffer (CmSi); Blue line: measurement in H2O buffer. The data underlying this Figure can be found in S1 Data. (TIFF) [file pbio.3003080.s005.tiff]

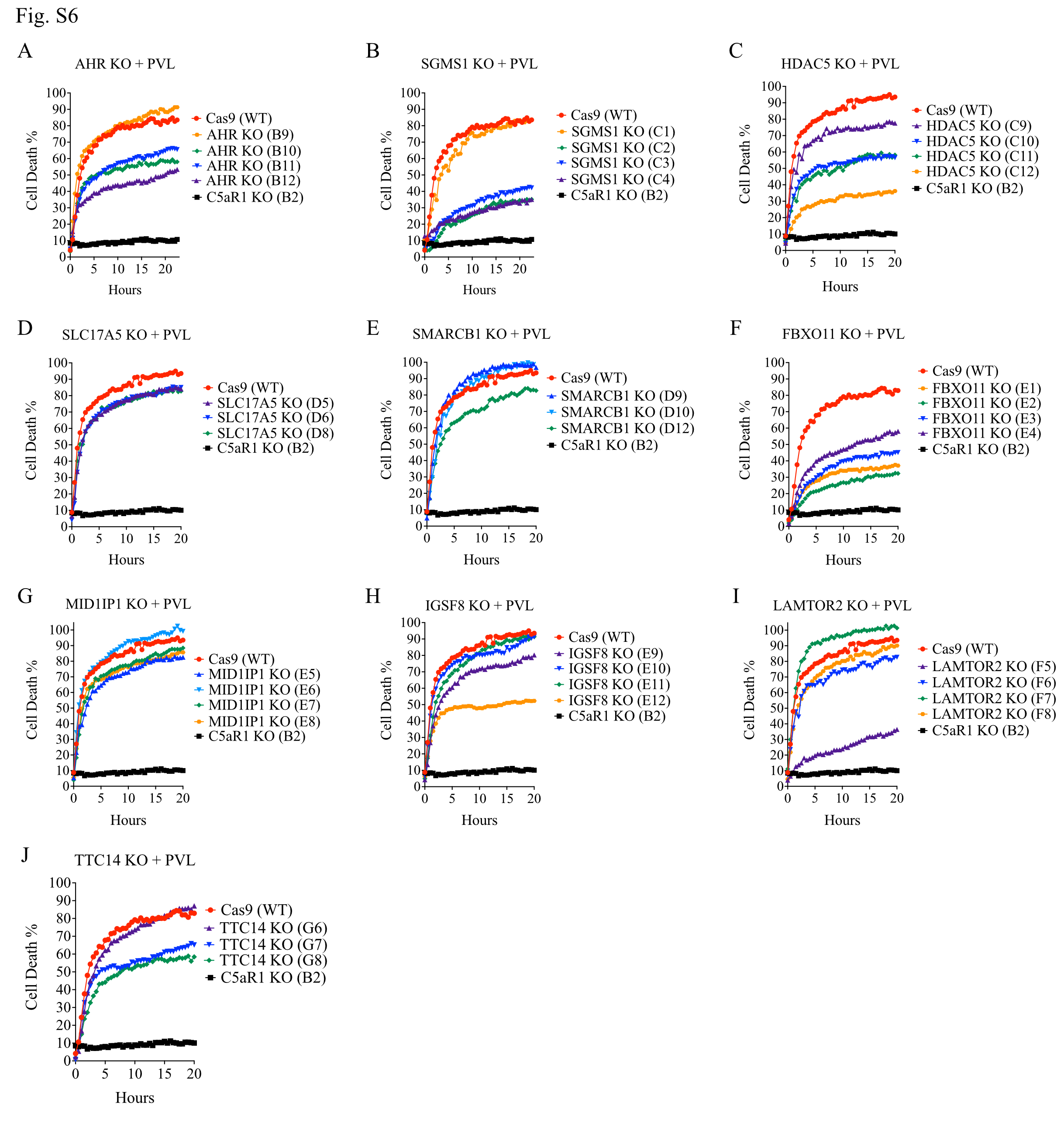

Supplement: S6 Fig — Cell death (Draq7-positive) of wild type, homogenous C5aR1 knockout and 4 independent heterogenous THP-1 macrophages targeting (A) AHR, (B) SGMS1, (C) HDAC5, (D) SLC17A5, (E) SMARCB1, (F) FBXO11, (G) MID1IP1, (H) IGSF8, (I) LAMTOR2, and (J) TTC14 treated with PVL overtime. The data underlying this Figure can be found in S1 Data. (TIFF) [file pbio.3003080.s006.tiff]

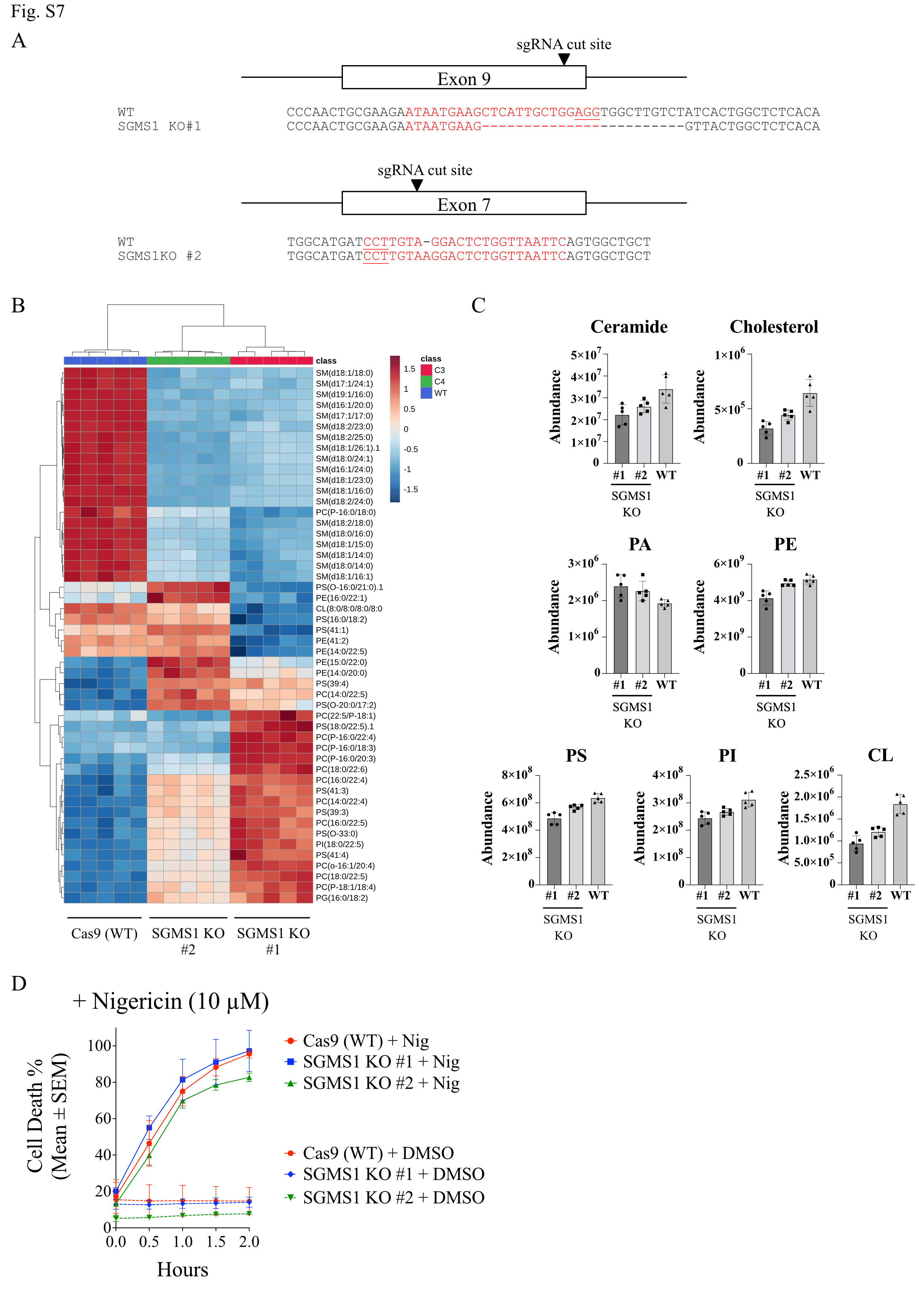

Supplement: S7 Fig — (A) Genomic sequence of sgRNA targeting site for two independent clones of SGMS1 knockout THP-1 cells. (B–C) Clustered heatmap (B) and relative abundance levels (C) of lipids in wild type and SGMS1 KO clones #1 and #2. Phosphatidic acid (PA), phosphatidylethanolamine (PE), phosphatidylserine (PS), phosphatidylinositol (PI) and cardiolipin (CL). (D) Cell death (Draq7-positive) of Cas9-expressing WT or SGMS1 knockout THP-1 macrophages treated with nigericin or DMSO overtime. Mean ± SEM from three independent experiments shown. The data underlying this Figure can be found in S1 Data. (TIFF) [file pbio.3003080.s007.tiff]

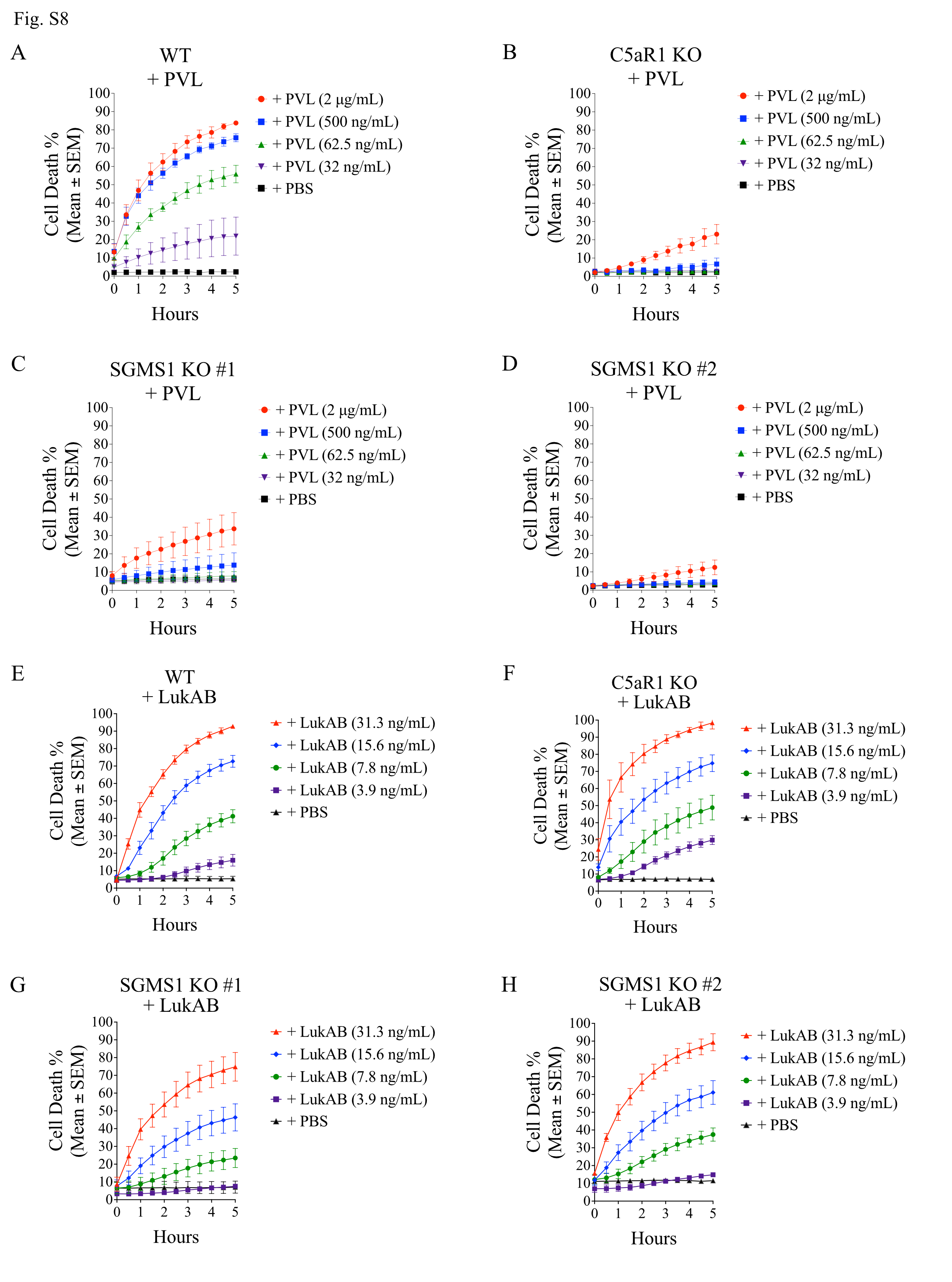

Supplement: S8 Fig — (A–H) Cell death (Draq7-positive) of wild type, SGMS1 KO or C5aR1 KO THP-1 macrophages treated with different concentrations of (A–D) purified recombinant PVL (32, 62.5, 500, 2,000 ng/ml) or (E–H) purified recombinant LukAB (3.9, 7.8, 15.6, or 31.3 ng/ml) overtime. Mean ± SEM from three independent experiments shown. The data underlying this Figure can be found in S1 Data. (TIFF) [file pbio.3003080.s008.tiff]

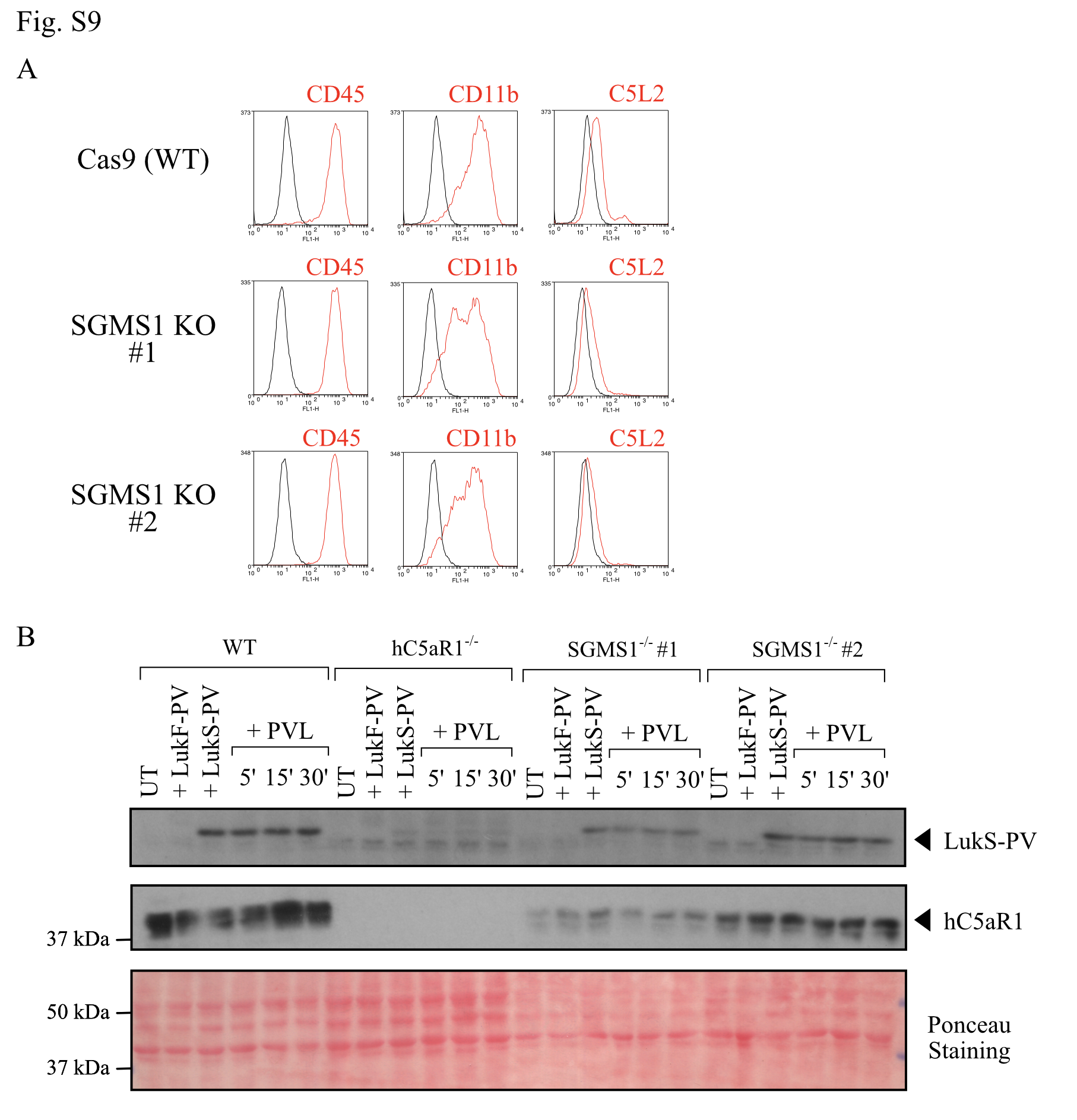

Supplement: S9 Fig — (A) Cell-surface levels of CD45, CD11b and C5L2 (C5aR2) in Cas9-expressing THP-1 derived macrophages and two independent clones of SGMS1 knockout macrophages using flow cytometry. Data representative of three independent experiments. (B) Total cell lysates of WT, C5aR1 and SGMS1 KO THP-1 macrophages treated with PVL for 5, 15, and 30 min were probed with antibodies against LukS-PV and C5aR1. Untreated (UT) and recombinant LukF-PV and LukS-PV were included as controls. Ponceau staining was used as loading control. Data representative of three independent experiments. (TIFF) [file pbio.3003080.s009.tiff]

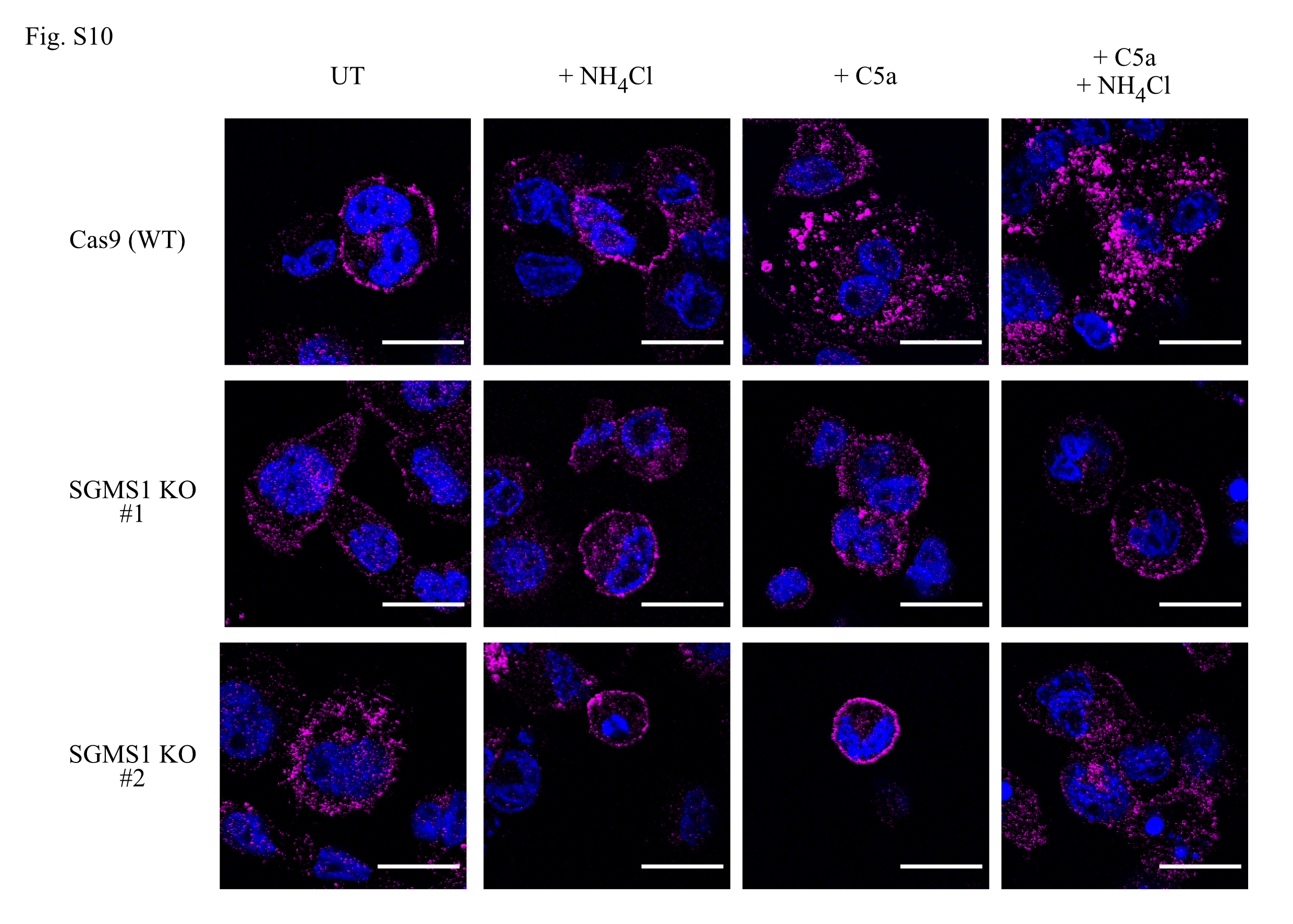

Supplement: S10 Fig — THP-1 macrophages were pretreated with cycloheximide with/without ammonium chloride for 1 h prior to C5a treatment for 3 h and then probed with antibody against C5aR1 (magenta). Nuclei are stained with DAPI (blue). Scale bar is 20 µm. (TIFF) [file pbio.3003080.s010.tiff]

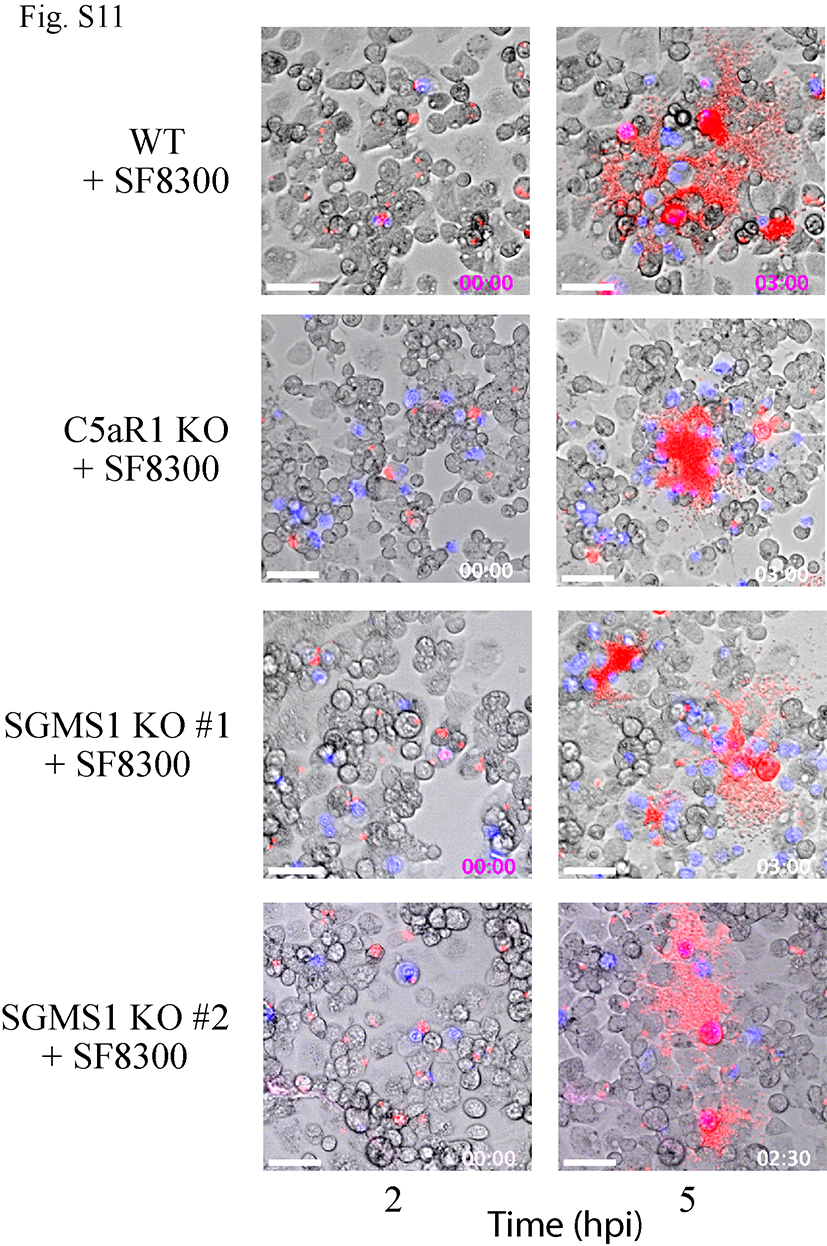

Supplement: S11 Fig — Images from live-cell video microscopy of WT, C5aR1 and SGMS KO THP-1 macrophages infected with DsRed-expressing S. aureus (USA300, SF8300 clone, red) in the presence of Draq7 (blue, dead cells) at 2 and 5 h post infection. Scale bar is 50 μm; Images representative of three independent experiments. (TIF) [file pbio.3003080.s011.tif]

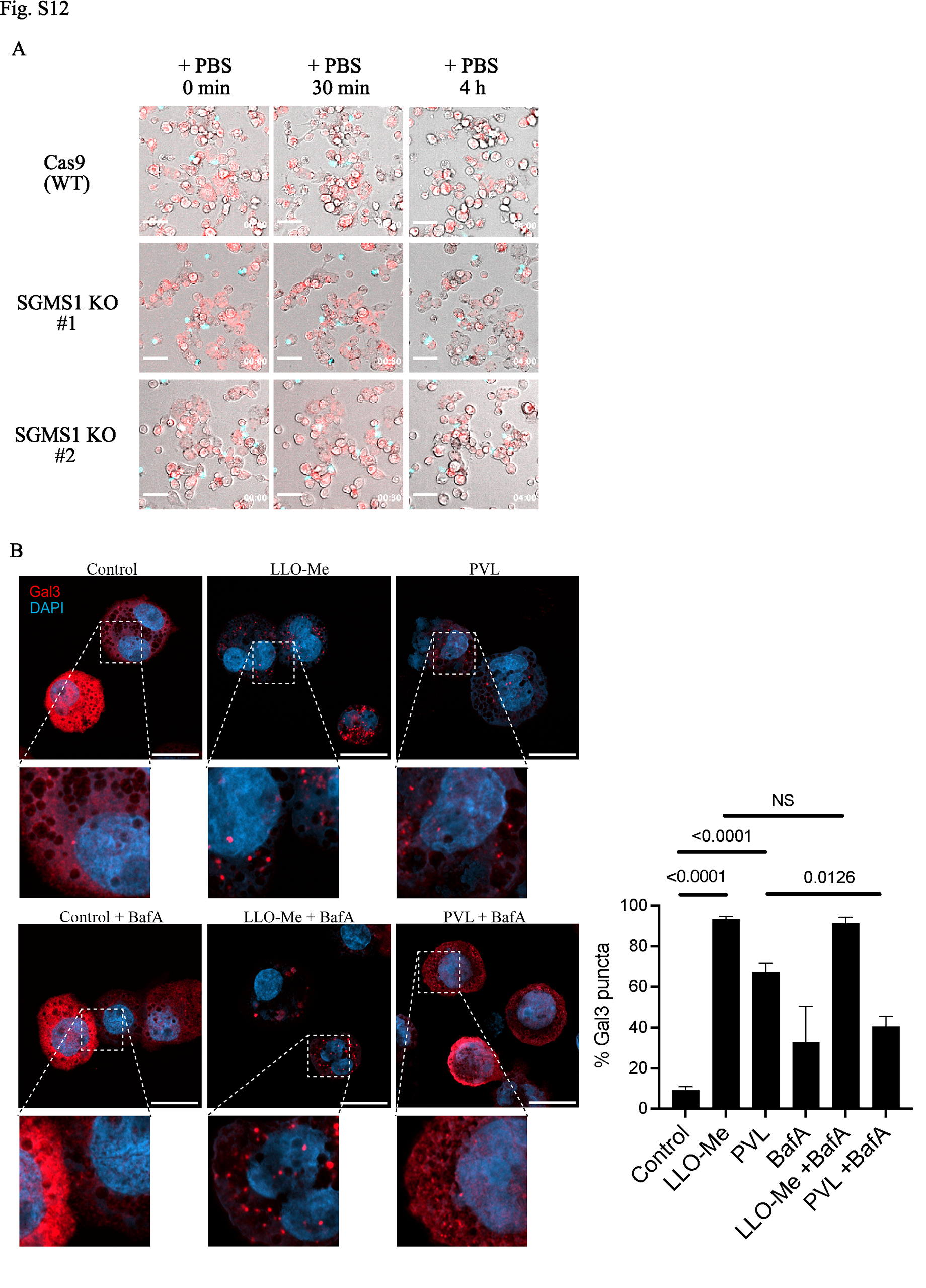

Supplement: S12 Fig — (A) Lysotracker Red stained wild type or SGMS1 KO THP-1 macrophages were treated with PBS in the presence of Draq7 (blue, dead cells) for indicated time. Scale bar is 50 μm; Data representative of three independent experiments. (B) Galectin-3 staining (red) of THP-1 macrophages treated with LLO-Me (500 µM), PVL (62.5 ng/ml) or control vehicle with or without Bafilomycin A1 (BafA, 100 nM) for 1 h. Nuclei were stained with DAPI (blue). Scale bar is 20 µm. Cells containing Galectin-3 puncta (% Gal3 puncta) were quantified from >50 individual cells per experiments. Mean, SEM, and P-values from three independent experiments shown. The data underlying this Figure can be found in S1 Data. (TIF) [file pbio.3003080.s012.tif]

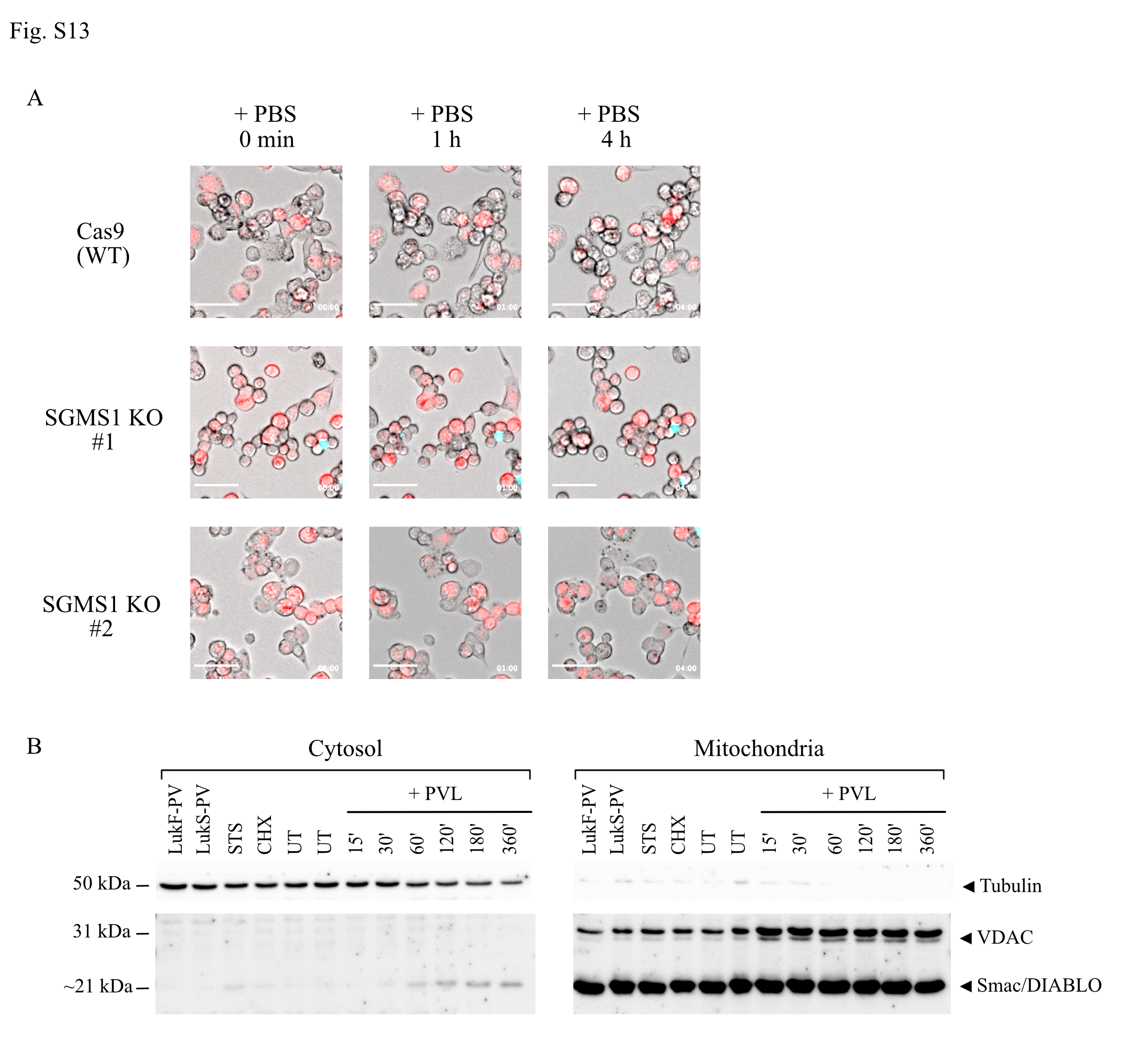

Supplement: S13 Fig — (A) Single-cell analysis of MitoTracker TMRM stained (red) wild type (Cas9 WT) or SGMS1 KO THP-1 macrophages treated with PBS in the presence of Draq7 (blue, dead cells) for indicated time. Scale bar is 50 μm; Data representative of three independent experiments. (B) THP-1 macrophages were treated with PVL for the indicated times, subunit LukS-PV, LukF-PV, and the apoptosis inducing compounds staurosporine (STS) and cycloheximide (CHX) for 6 h or left untreated (UT). The cytosolic and mitochondrial fractions were probed with antibodies against Smac/DIABLO, VDAC (mitochondrial protein) and tubulin (loading control). (TIFF) [file pbio.3003080.s013.tiff]

Fig. S14

A

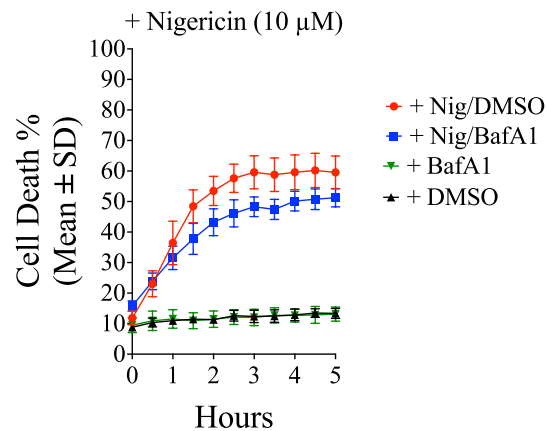

B

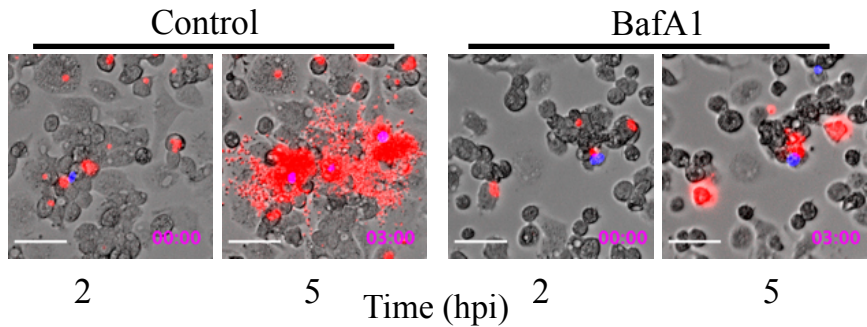

C

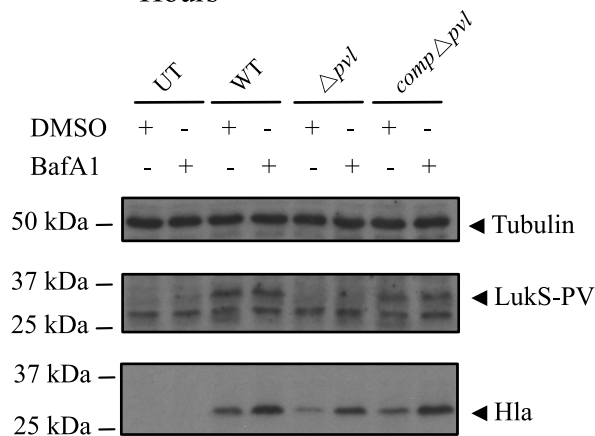

D

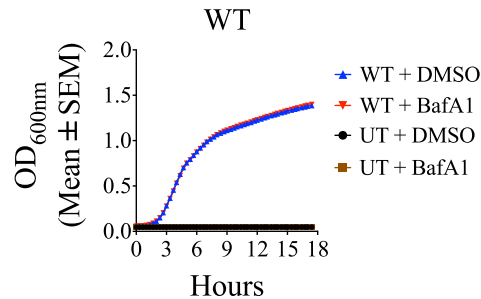

E

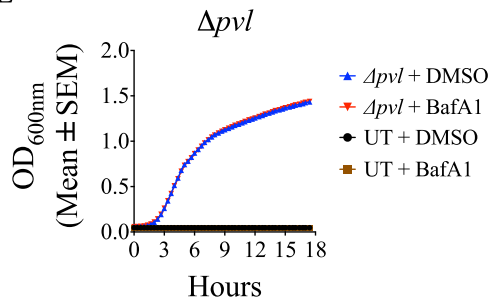

F

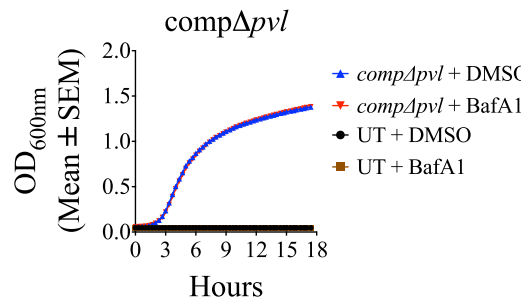

Supplement: S14 Fig — (A) Cell death (Draq7-positive) of THP-1 macrophages treated with nigericin (Nig), and Bafilomycin A1 (BafA1) or DMSO overtime. Mean +/- SEM from three independent experiments shown. (B) THP-1 macrophages infected with DsRed-expressing WT S. aureus (MOI = 10) in the presence of Bafilomycin A1 (BafA1) or vehicle control were analyzed by time-lapse imaging. Images are from 2 and 5 h post infection. Draq7 staining was included to identify dead cells. (C) THP-1 macrophages were treated with BafA1 or DMSO after infection with S. aureus WT, Δpvl and complemented strain (compΔpvl) for 4 h post infection. Total cell lysates were probed with antibodies against LukS-PV, a-hemolysin (Hla, control for S. aureus infections), and tubulin (loading control for macrophages). (C–E) Growth curve of S. aureus (C) WT, (D) Δpvl, and (E) compΔpvl in broth media with BafA1 or DMSO over 18 h. Untreated (UT) media without bacteria are controls Mean from three independent samples. The data underlying this Figure can be found in S1 Data. (PDF) [file pbio.3003080.s014.pdf]

Fig 1A

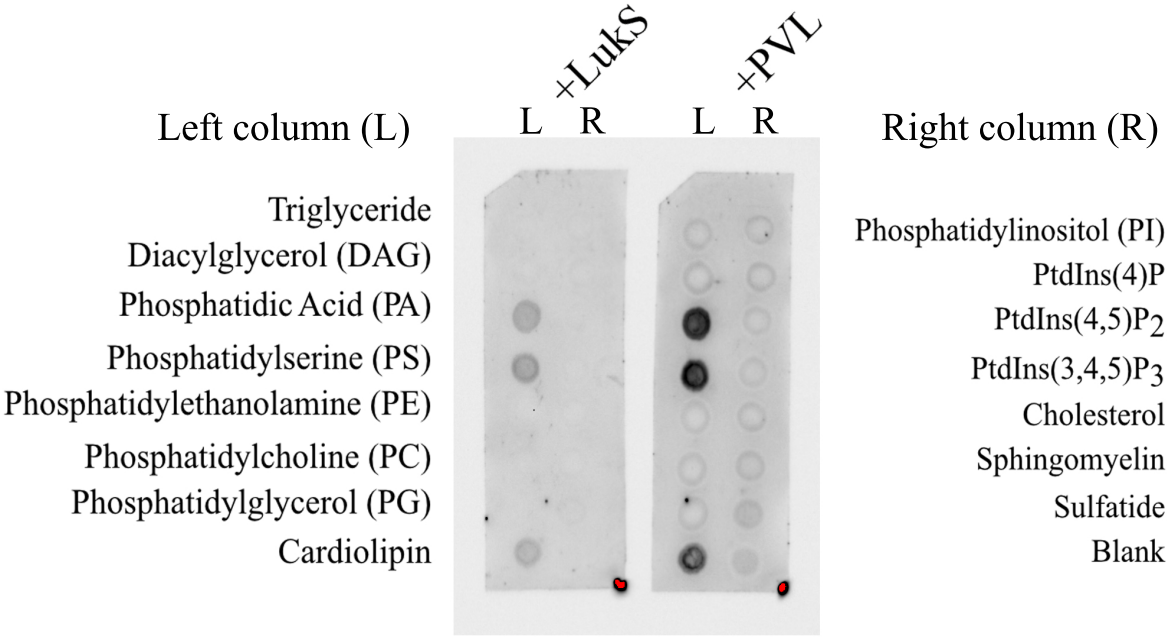

S1 Fig C

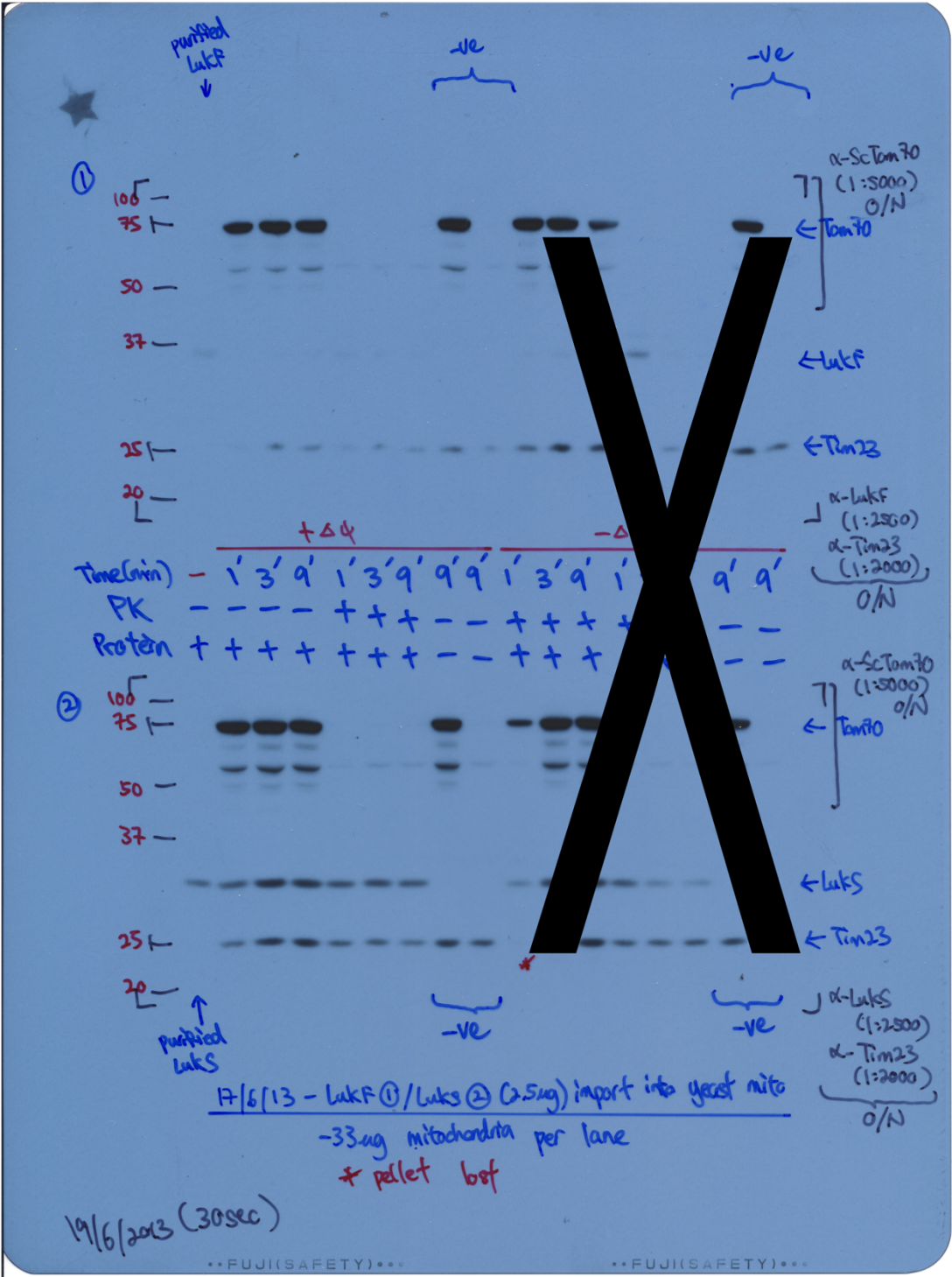

S9 Fig B

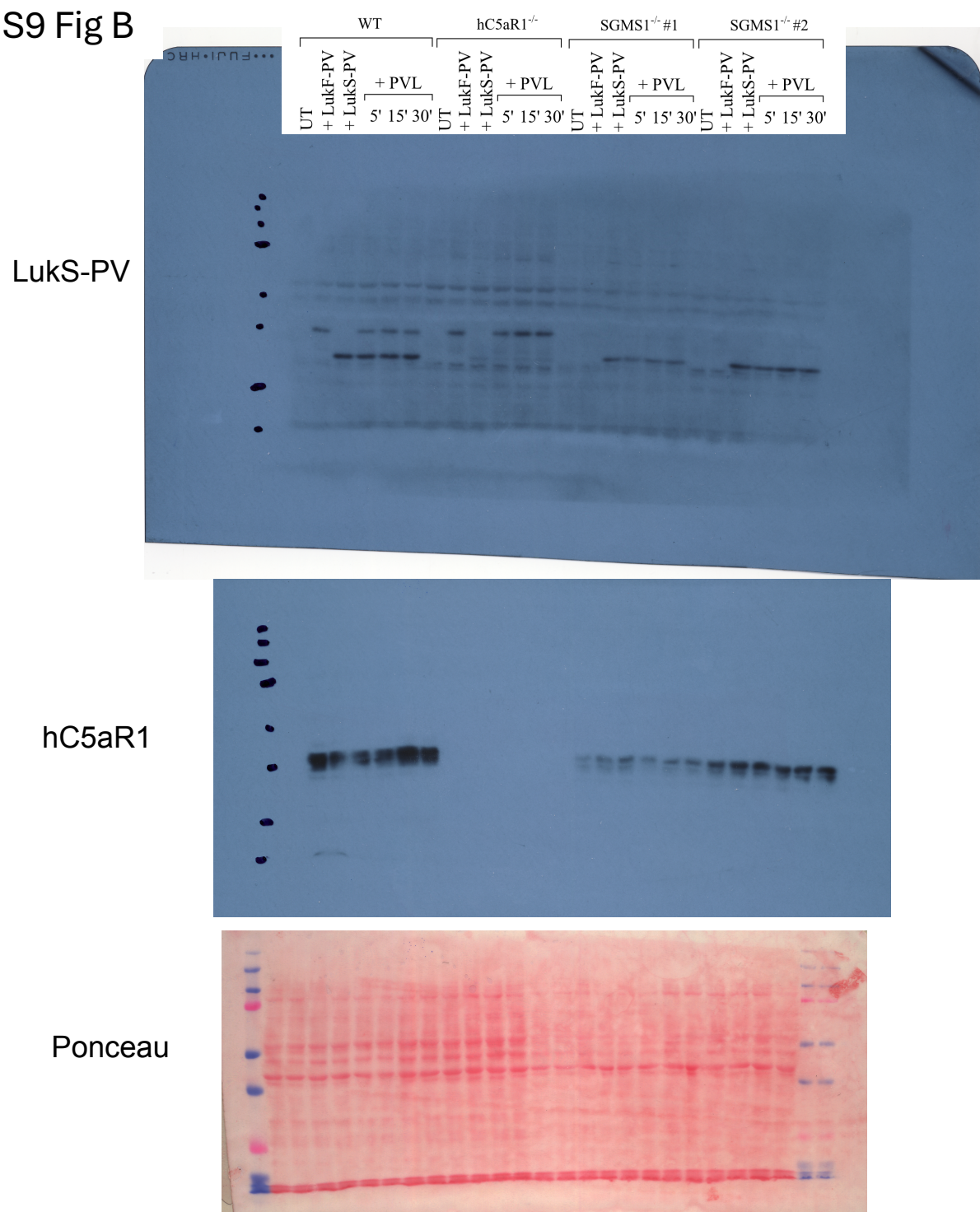

S13 Fig B

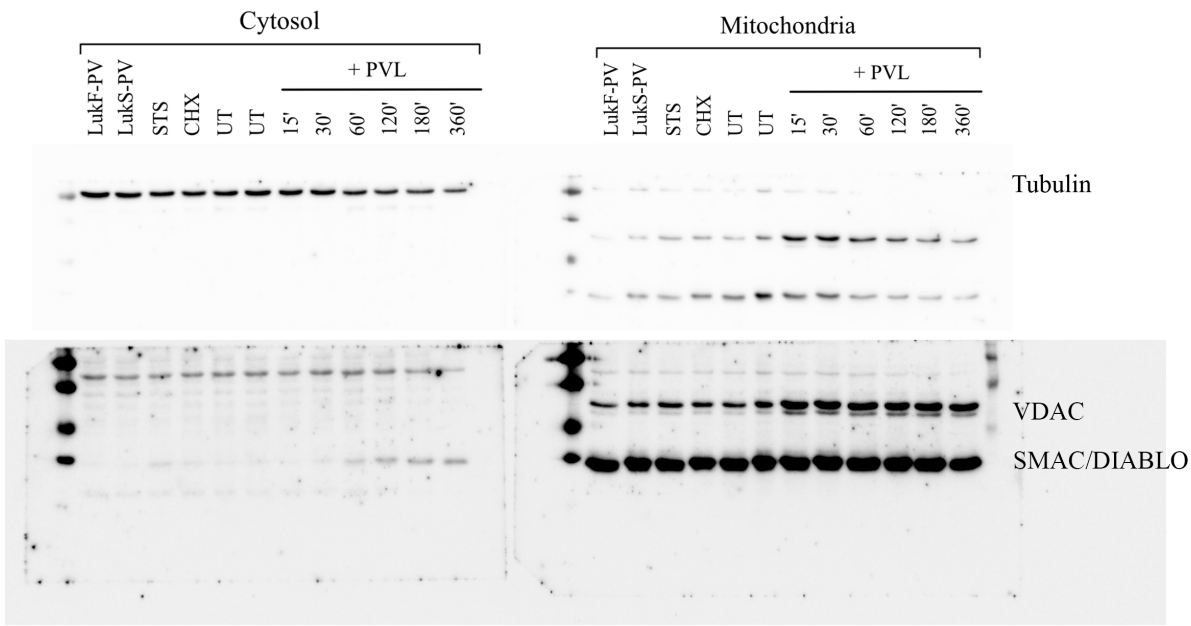

S14 Fig C

LukS-PV

Hla

Tubulin

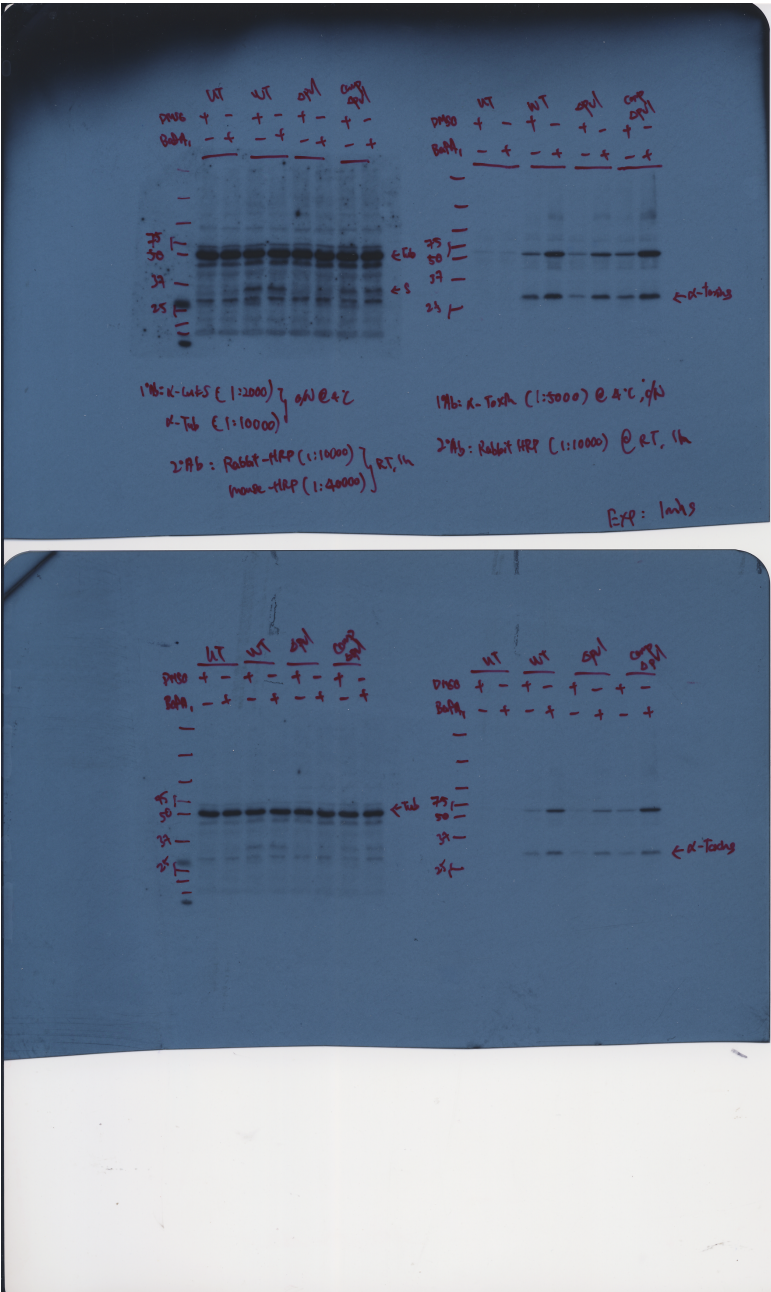

Supplement: S1 Raw Images — (PDF) [file pbio.3003080.s025.pdf]

Fig 2D

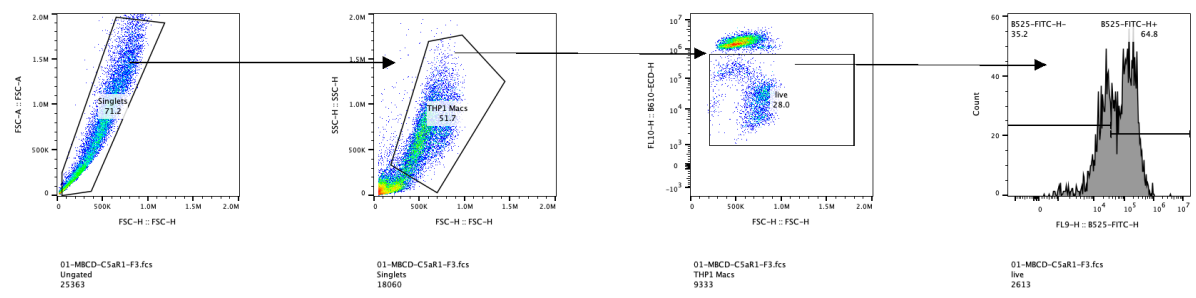

Fig 3A

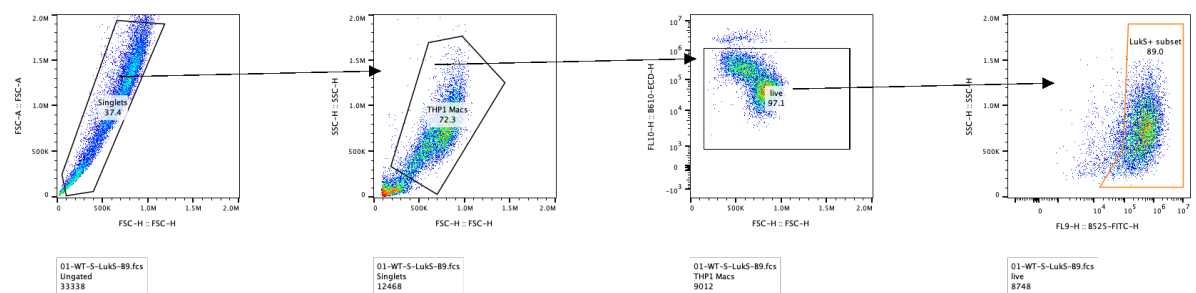

Figure S9A

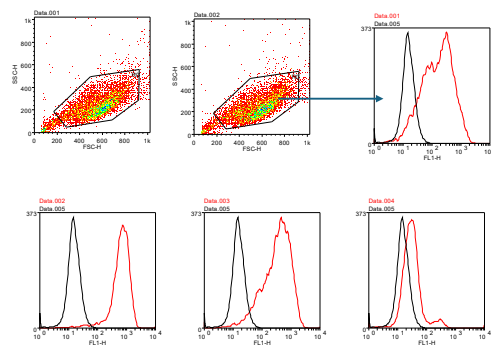

Supplement: S2 Data — (PDF) [file pbio.3003080.s033.pdf]
