# Supplementary material for: Staphylococcal toxin PVL ruptures model membranes under acidic conditions through interactions with cardiolipin and phosphatidic acid
Source: PLoS Biol. 2025 Apr 15;23(4):e3003080. doi: 10.1371/journal.pbio.3003080 (PMC12052211; doi:10.1371/journal.pbio.3003080)
Supplement: S2 Table — (DOCX) [file pbio.3003080.s027.docx]

Table S2. The theoretical neutron scattering length density (SLD) of lipids and PVL.

| **Component** | **Neutron scattering length density (SLD) (ρ) (× 10^-6^Å^-2^) ^[a]^** |
| --- | --- |
| 10 mM HEPES/5 mM CaCl_2_/150 mM NaCl pD 7.4 D_2_O buffer | 6.35 |
| 10 mM HEPES/5 mM CaCl_2_/150 mM NaCl pD/pH 7.4 CmSi buffer^[b]^ | 2.07 |
| 10 mM HEPES/5 mM CaCl_2_/150 mM NaCl pH 7.4 H_2_O buffer | -0.56 |
| Citrate-phosphate D_2_O buffer, pD 5.0 | 6.35 |
| Citrate-phosphate CmSi buffer, pH/pD 5.0 | 2.07 |
| Citrate-phosphate H_2_O buffer, pH 5.0 | -0.56 |
| Silicon | 2.07 |
| Silicon oxide (SiO_2_) | 3.47 |
| POPC/TOCL (w/w 2:1) head group in D_2_O | 2.13 |
| POPC/TOCL (w/w 2:1) head group in CmSi | 2.10 |
| POPC/TOCL (w/w 2:1) head group in H_2_O | 2.09 |
| POPC/TOCL (w/w 2:1) lipid tails in D_2_O/CmSi/H_2_O | -0.28 |
| POPC/POPA (w/w 2:1) head group in D_2_O | 2.94 |
| POPC/POPA (w/w 2:1) head group in CmSi | 2.79 |
| POPC/POPA (w/w 2:1) head group in H_2_O | 2.70 |
| POPC/POPA (w/w 2:1) lipid tails in D_2_O/CmSi/H_2_O | -0.28 |
| PVL in D_2_O | 3.53 |
| PVL in CmSi | 2.59 |
| PVL in H_2_O | 2.01 |

^[a]^SLDs for PVL were calculated based on the volumes of the amino acids sequences. ^[b]^CmSi: Silicon-matched water, which contains 38% D_2_O and 62% H_2_O.
