# Supplementary material for: Staphylococcal toxin PVL ruptures model membranes under acidic conditions through interactions with cardiolipin and phosphatidic acid
Source: PLoS Biol. 2025 Apr 15;23(4):e3003080. doi: 10.1371/journal.pbio.3003080 (PMC12052211; doi:10.1371/journal.pbio.3003080)

Table S3. Fitted thickness and the calculated volume fractions of POPC/TOCL bilayer in the NR study with the binding of PVL in (A) pH 5.0 and (B) pH 7.4.

(A)

| **Layer^[a]^** | **Component** | **Thickness (Å)** | **% Lipid** | **% PVL** | **% Water** |
| --- | --- | --- | --- | --- | --- |
| 1 | SiO_2_ | 10.0 | NF | NF | NF |
| 2 | Phospholipid head (IH) | 11.6 ± 0.9 | 57.8 ± 3.4 | NF | 42.2 ± 3.4 |
| 3 | Phospholipid tail | 29.5 ± 1.2 | 95.5 ± 2.7 | 2.5 ± 1.1 | 2.1 ± 1.6 |
| 4 | Phospholipid head (OH) | 7.3 ± 1.2 | 50.9 ± 7.9 | 17.7 ± 2.6 | 31.4 ± 10.4 |
| 5 | PVL | 70.9 ± 2.0 | NF | 16.1 ± 0.4 | 83.9 ± 0.4 |
| 6 | Lipid | 32.9 ± 1.4 | 22.9 ± 3.0 | NF | 77.1 ± 3.0 |

^[a]^Roughness of each layer was fitted at 4.0 Å. NF: not found.


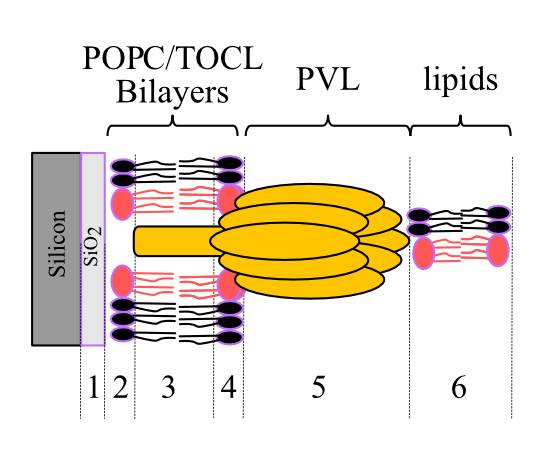


(B)

| **Layer^[a]^** | **Component** | **Thickness (Å)** | **% Lipid** | **% PVL** | **% Water** |
| --- | --- | --- | --- | --- | --- |
| 1 | SiO_2_ | 6.95 | NF | NF | NF |
| 2 | Phospholipid head (IH) | 11.1 ± 1.9 | 49.1 ± 5.1 | NF | 50.9 ± 5.1 |
| 3 | Phospholipid tail | 31.2 ± 1.1 | 94.9 ± 3.3 | NF | 5.1 ± 3.3 |
| 4 | Phospholipid head (OH) | 8.0 ± 2.0 | 48.5 ± 6.7 | 1.4 ± 0.3 | 50.2 ± 6.7 |
| 5 | PVL | 34.4 ± 2.3 | NF | 2.0 ± 0.5 | 98.1 ± 0.5 |

^[a]^Roughness of each layer was fitted at 4.0 Å. NF: not found.


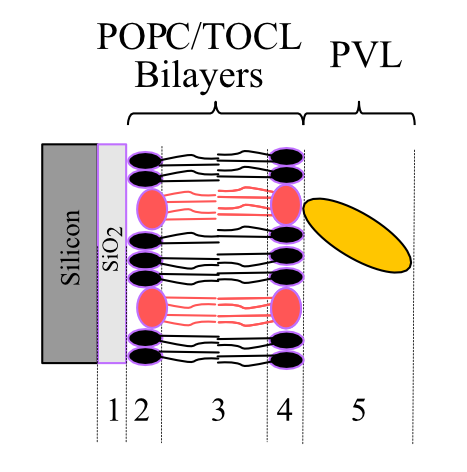

Supplement: S3 Table — (DOCX) [file pbio.3003080.s028.docx]
