# Supplementary material for: Staphylococcal toxin PVL ruptures model membranes under acidic conditions through interactions with cardiolipin and phosphatidic acid
Source: PLoS Biol. 2025 Apr 15;23(4):e3003080. doi: 10.1371/journal.pbio.3003080 (PMC12052211; doi:10.1371/journal.pbio.3003080)

Table S4. Fitted thickness and the calculated volume fractions of POPC/POPA bilayer in the NR study with the binding of PVL in (A) pH 5.0 and (B) pH 7.4.

(A)

| **Layer^[a]^** | **Component** | **Thickness (Å)** | **% Lipid** | **% PVL** | **% Water** |
| --- | --- | --- | --- | --- | --- |
| 1 | SiO_2_ | 12.5 ± 1.8 | NF | NF | NF |
| 2 | Phospholipid head (IH) | 8.3 ± 1.3 | 48.9 ± 1.3 | NF | 51.2 ± 1.3 |
| 3 | Phospholipid tail | 32.0 ± 3.9 | 94.9 ± 3.5 | 2.1 ± 0.9 | 3.0 ± 2.9 |
| 4 | Phospholipid head (OH) | 7.1 ± 0.5 | 47.0 ± 2.2 | 24.3 ± 0.4 | 28.7 ± 2.2 |
| 5 | Phospholipid head (IH)  + PVL | 7.9 ± 0.8 | 8.9 ± 1.6 | 25.9 ± 1.1 | 65.2 ± 2.6 |
| 6 | Phospholipid tail  + PVL | 30.3 ± 1.9 | 8.5 ± 2.9 | 23.6 ± 3.8 | 68.0 ± 6.1 |
| 7 | Phospholipid head (OH) + PVL | 8.7 ± 2.0 | 12.3 ± 3.1 | 24.8 ± 0.3 | 62.9 ± 3.4 |
| 8 | PVL | 21.2 ± 2.5 | NF | 28.0 ± 4.4 | 72.0 ± 4.4 |
| 9 | Lipid | 35.8 ± 2.8 | 28.8 ± 3.7 | NF | 71.2 ± 3.7 |
| 10 | PVL | 74.2 ± 2.0 | NF | 8.6 ± 1.4 | 91.4 ± 1.4 |
| 11 | Lipid | 39.0 ± 2.0 | 11.6 ± 0.7 | NF | 88.4 ± 0.7 |

^[a]^Roughness of each layer was fitted at 8.0 Å. NF: not found.


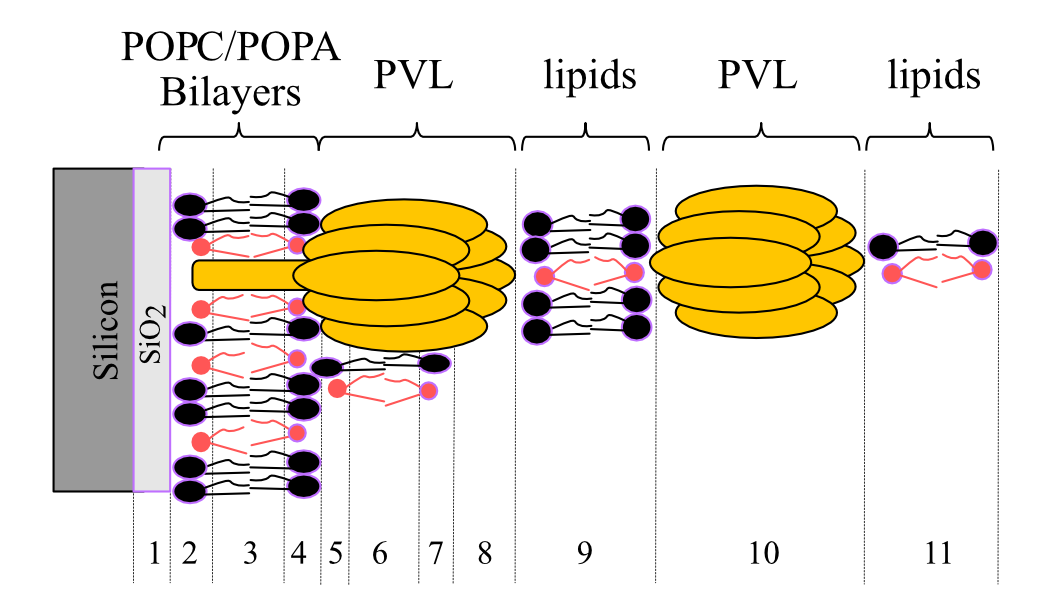


(B)

| **Layer^[a]^** | **Component** | **Thickness (Å)** | **% Lipid** | **% PVL** | **% Water** |
| --- | --- | --- | --- | --- | --- |
| 1 | SiO_2_ | 10.284 ± 0.5 | NF | NF | NF |
| 2 | Phospholipid head (IH) | 12.8 ± 0.8 | 60.0 ± 4.2 | NF | 40.1 ± 4.2 |
| 3 | Phospholipid tail | 31.7 ± 0.1 | 98.0 ± 1.9 | NF | 2.0 ± 1.9 |
| 4 | Phospholipid head (OH) | 7.3 ± 0.2 | 59.4 ± 2.8 | 2.4 ± 0.2 | 38.2 ± 3.0 |
| 5 | Phospholipid head (IH)  + PVL | 16.0 ± 0.9 | 1.6 ± 0.1 | 2.3 ± 0.1 | 96.2 ± 5.1 |
| 6 | Phospholipid tail  + PVL | 35.3 ± 2.4 | 1.7 ± 0.2 | 3.6 ± 0.5 | 94.7 ± 0.3 |
| 7 | Phospholipid head (OH)  + PVL | 16.3 ± 0.7 | 3.0 ± 0.1 | 3.1 ± 0.3 | 93.9 ± 1.2 |
| 8 | Lipid | 41.3 ± 1.5 | 4.7 ± 0.5 | NF | 95.3 ± 0.5 |

^[a]^Roughness of each layer was fitted at 4.0 Å. NF: not found.


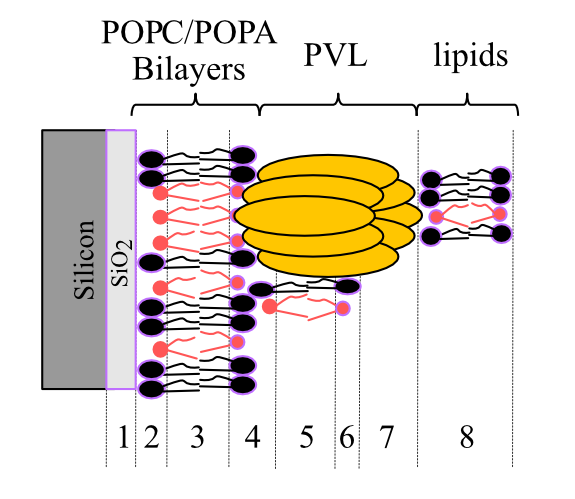

Supplement: S4 Table — (DOCX) [file pbio.3003080.s029.docx]
