# Supplementary material for: Staphylococcal toxin PVL ruptures model membranes under acidic conditions through interactions with cardiolipin and phosphatidic acid
Source: PLoS Biol. 2025 Apr 15;23(4):e3003080. doi: 10.1371/journal.pbio.3003080 (PMC12052211; doi:10.1371/journal.pbio.3003080)
Supplement: Attachment — Submitted filename: response_reviewers.docx [file pbio.3003080.s036.docx]

Our response to each point raised by the reviewers is highlighted in blue.

------------------------------------
REVIEWERS' COMMENTS:
------------------------------------


Reviewer #1: 

I have read the paper, the comments of the referees of the previous version and the response of the authors on their suggestions. In all, authors have addressed many different issues but for me the major issues have not been dealt with yet.

My major concern remains the concentration used. The authors claim that their high concentration can be observed in vivo, but that is not the issue. The issue is what is responsible for killing at the site of infection there is a halo of toxin surrounding viable bacteria, and cells (neutrophils and macrophages with the appropriate set of receptors) approach these bacteria and will be promptly killed at low concentrations (ng range), way before they reach the concentration of toxin used in these studies with liposomes (low to high ug range). So in my mind, the phenomena studied in this paper are not of importance for neutrophils and macrophages. They could be of importance in non-phagocytic cell (lacking the C5aR and CD45). The main question is how important that is in the pathophysiology of S. aureus infection. Also, because of the same reasons the title of the paper is highly confusing.

Although some of the experiments are impressive and well performed, the issue as illustrated above is not with the execution of the experiments but with the questions the authors pose and with the conclusions they draw. Overall the answers to the referees comments (i agree with all of the three referees) are not convincing to me.

We have amended the title to more closely reflect our main findings. To reiterate, in contrast to previous studies, the current manuscript has identified conditions that allow high concentrations of PVL to rupture model membranes.

We also have discussed now the shortcomings and limitations of the study and emphasized that high toxin concentrations were used to detect the lipid interactions on artificial membranes. We have stated that during lung infections the novel PVL-lipid interaction may not directly apply to macrophages and other innate immune cells that are killed at low concentrations, but may be more applicable to non-phagocytic cells that are deficient in toxin receptors. Overall, we conclude that more work is required to validate our findings in more physiological settings.

------------------------------------


Reviewer #2: 

In their manuscript, Chow and colleagues nicely demonstrated the lipid binding partners of  PVL in membranes, the requirement for sphingomyelin and cholesterol for internalization, and low pH for permeabilization of lysosome. They then performed S. aureus infection experiments with WT and PVL mutant, and bafilomycin to verify the role of PLV and low pH in S. aureus infectious physiology.  This aspect of the study, even with the added data, still require more rigor.

Major comments
1) Sphingomyelin breakdown product ceramide is a second messenger in apoptotic cascade activation.  Hence, KO of sphingomyelin could reduce cell death independently of its role in trafficking.  Interpretation of findings linked to SGMS1 KO need to take that into account.

We appreciate that ceramide can signal to the apoptotic and pyroptotic machinery. PVL kills THP macrophages by activating NLRP3-dependent pyroptosis (PMID: 32531864). Nigericin (a canonical NLRP3 activator) kills sphingomyelin deficient THP macrophages normally (Fig S7D). Also, there was no statistical difference in ceramide levels between WT and SGMS1 KO macrophages (Fig S7C). We therefore concluded that difference in cell death rates between WT and SGMS1 KO macrophages exposed to PVL are not directly related to defects in apoptosis and pyroptosis.

We have included in the revised discussion the possibility that SGMS1 contributes to PVL activity by regulating cell death signalling.

2) Although high concentration of PVL is measured in human samples, findings made on artificial membranes using higher PVL doses may not be relevant to what actually happens in the THP or standard macrophage assays, and this should be acknowledged by the authors.

We have now expanded the last paragraph in the discussion on the limitations of the study. We highlight that high concentration of PVL were used on artificial membranes and caution that our findings may not directly relate to cultured macrophages.

3) Figure 6B and E.  The authors propose that PVL should rupture the lysosome at lower pH and drive egress of S. aureus into the cytosol.  Once in the cytosol, S. aureus should overgrowth (based on the video).  Yet they state that there is no difference in intracellular CFU between WT and delta PVL (C), or control or bafilomycin (E). This does not fit the data.
Visually, it does look as if there is a difference between WT and delta PVL (C) and control versus bafilomycin (E) even is there is not noted statistical significance. Lack of significance could be attributed to lack of power.  However, if that is the case,  increased extracellular CFUs could simply be a reflection of increased intracellular CFUs. Irrespective, the experiment setup is not adequate to discriminate and verify the proposed hypothesis.
To fully test the hypothesis, the Bafilomycin experiment should really be performed using both WT and PVL KO  to investigate if acidification related the  findings (CFU and macrophage cell death) are directly related to PVL. In addition to measuring CFUs and (macrophage) cell deaths, the authors should quantify permeabilization of lysosome and mitrochondria in this experiment.

We have now included new data (Fig 6B and C), including Bafilomycin A1 treatment of WT and PVL KO. As suggested, we have focused on intracellular bacterial numbers to test whether loss of PVL affects intracellular S. aureus numbers. After nine independent repeats, we did not detect a significant difference in intracellular CFUs between WT and PVL KO (Fig 6B). There was, however, a significant difference between control and Bafilomycin A treatment in WT, and also in PVL KO infected macrophages.

We also addressed whether Bafilomycin A treatment affects the ability of PVL KO to escape from infected macrophages. As observed previously, loss of PVL or Bafilomycin A treatment reduced extracellular bacterial numbers (Fig 6C). Bafilomycin A treatment also affect extracellular PVL KO numbers, but this was not statistically significant (even when potential outliers in the control group are excluded). We note that the difference between WT and PVL KO is only 2-3 fold, which is lost at later time points as bacteria assume rapid growth in culture media. As supported by the imaging, loss of PVL or Bafilomycin A treatment transiently reduces the ability of S. aureus to escape from macrophages (Fig 6D). This appears to be independent of the intracellular numbers as we did not detect difference between WT and PVL KO (Fig 6B). Rather, extracellular numbers are associated with macrophage survival rates, which are somewhat delayed in PVL KO or Bafilomycin A treatment.

It is clear, however, that other mechanisms enable S. aureus to escape from macrophages. This may include additional toxins that are pH sensitive. Based on the imaging (and partly CFUs), we also noted that Bafilomycin A further dampened escape of PVL KO from infected macrophages. If PVL promotes escape depending on acidic environments, we would expect no difference. It is possible then that Bafilomycin A1 lysosome-independent mechanisms contribute under these conditions, as BafA1 may have additional effects. Alternatively, PVL may further contribute to S. aureus escape depending on neutral pH conditions. Regardless, it seems that BafA1 and PVL have additive effects, but we do not know why. We also aimed to stain mitochondria and lysosome in bacterial infected macrophages to quantify organelle permeabilization. However, we have not been able to obtain consistent results, likely reflecting cross-staining with bacteria and heterogenous responses (as not all infected macrophages respond at the same time). Future work is now focused on obtaining high-res single cell data, which is beyond the scope of this study.

We have discussed these possibilities in the revised discussion.

4) The title of the paper implies that PVL lysis of macrophages depends on PA, cardiolipin and acidic environment.  Demonstration of these PVL functions in the context of a S. aureus infection is currently not robust.

We have amended the title to reflect that our findings are supported using recombinant PVL.

Minor comments
1) Concentration of purified toxins and S. aureus to host cell MOI used in assays should be noted in the figure legends.

We have now included toxin concentrations and MOI used in the figure legends.

4) The level of LukS-PV (MFI) bound to SGMS1 KO is half that bound to WT macrophages.  While that likely does not substantially impact the conclusion drawn on the role of sphingomyelin in PVL trafficking, it is unclear if that would affect conclusion drawn on cell death if sphingomyelin is involved in cell death.  This should be discussed.

We have discussed in the revised manuscript the possibility that the reduced binding of PVL to SGMS1 KO macrophages may contribute to the resistance of these cell to the toxin via sphingolipid-mediated cell death signalling.

4) I agree that in absence of KO in host, it would be difficult to confirm the role of specific lipids in PVL interaction in SA infection.  This has been noted as a limitation in the discussion.

------------------------------------


Reviewer #3:

I consider that Chow et al. have addressed the main concerns raised by the reviewers during the initial consideration. Consequently, I find that the manuscript now fits well within the scope of the current forum.

However, I would like to point out one critical experiment that was not previously raised by the reviewers. It is standard practice in the field, to evaluate endolysosomal rupture using well-characterized markers such as **Galectins (e.g., Galectin-3**, Galectin-8) and the recruitment of endomembrane damage repair machinery (e.g., ESCRT, and now more recently PI4K2A, stress granules, etc.). Unfortunately, the current manuscript predominantly relies on Lysotracker images, which lack high resolution. Additionally, Lysotracker staining can be influenced by factors other than lysosomal membrane damage, such as pH neutralization or changes, which may occur independently of membrane damage. 

To strengthen the main findings, I recommend that the authors in corporate some of these standard studies, even if performed on fixed cells. For instance, studies have explored the link between endomembrane damage and mitochondrial function (10.1038/s41467-022-34632-8). Other relevant examples include: 10.1242/jcs.252973, 10.1016/j.devcel.2019.10.025, 10.7554/eLife.85727, 10.1038/s41586-023-06726-w, and 10.1371/journal.ppat.1007501. Incorporating such studies would significantly enhance the robustness and reliability of their conclusions. This would significantly enhance the robustness and reliability of their conclusions.

We have now performed Galectin-3 staining of THP-1 macrophages treated with PVL and LLO-Me (positive control). As shown in Figure S12B, PVL induced the formation of Galectin-3 puncta, which was sensitive to Bafilomycin A1 treatment. We believe these strengthen our main finding that PVL ruptures endolysosomal organelles.
