# Supplementary material for: Staphylococcal toxin PVL ruptures model membranes under acidic conditions through interactions with cardiolipin and phosphatidic acid
Source: PLoS Biol. 2025 Apr 15;23(4):e3003080. doi: 10.1371/journal.pbio.3003080 (PMC12052211; doi:10.1371/journal.pbio.3003080)
Supplement: Attachment — Submitted filename: Reviewer_comments_auresp_4.docx [file pbio.3003080.s038.docx]

Our response to the reviewers’ comments (blue):

Reviewer #3: 

Panel S12B now shows lysosome damage analysis using a GAL-3 puncta readout. One concern I have with the experiment is that LLOMe is a very well known cathepsin C substrate and, therefore, proteolytically activity-dependent. The use of an inhibitor of V-ATPase, such as bafilomycin, which will neutralize the lysosomes, should block its activity. However, the authors show no effect. Given the study's importance, the image resolution should be improved, ideally by showing multiple cells with a zoomed-in section to highlight the currently blurry puncta. The authors could enhance resolution by adjusting the acquisition parameters on their confocal system.

We appreciate the expertise from Reviewer 3 and the Academic Editor on this matter, as the analysis of lysosome damage by LLOMe is a comparatively new area to us. Because of this, we are chiefly guided by the literature. After re-reading the relevant papers, the molecular mechanisms by which LLOMe causes lysosome rupture and cell death are not fully understood. The current model states that LLOMe accumulates in lysosomes, which may or may not depend on acidic environments and/or hydrolases. The LLOMe derived leucine dipeptide causes intra-lysosomal osmotic pressure as shown over 40 years ago with other amino acid ester (DOI: 10.1016/0005-2736(73)90114-4), which leads to lysosomal membrane permeabilization, increased pH and loss of lysosomal activity.

Reviewer 3 states that “LLOMe is a very well known cathepsin C substrate and, therefore, proteolytically activity-dependent.” Cathepsin C is an amino-dipeptidase. It is thus unclear to us how a dipeptidase can be proteolytically active against the leucine dipeptide. Cathepsin C also contains acyl transferase activity and thus can transfer a dipeptidyl residue onto an amino group rather than water as occurs during proteolysis, particularly at increasing pH (PMID: 4306035). This reaction creates polypeptides including LLOMe polymers. LLOMe polymers are thought to damage membranes, but the molecular mechanism remains controversial. In the original paper from 1990 that demonstrates a role of cathepsin in LLOMe toxicity, Thiele and Lipsky noted that “levels of tritium in CC13COOH precipitates (which correlates with LLOMe toxicity) actually increased when NH4Cl was added at concentrations known to increase lysosomal pH and thereby inhibit many lysosomal proteases.” In other words, acidic pH is not required for LLOMe polymer formation. A more recent study showed that Bafilomycin A1 had little effect on LLOMe induced lysosomal disruption (doi: 10.1242/jcs.204529). As such, the current model predicts that LLOMe primarily causes increased lysosomal pH, which is instant as measured by rapid loss of LysoTracker staining and largely independent of cathepsin C. Typically, lysosomal membrane rupture, as detected by Gal3 puncta formation, occurs later at around 30 minutes post treatment, depending on high concentrations of LLOMe and cathepsin C, likely due to the formation of polymers. As such, Bafilomycin A1 should not affect LLOMe-dependent Gal3 puncta formation. We have verified our results by an independent laboratory, which also concluded that Bafilomycin A1 had little effect on LLOMe-dependent Gal3 puncta formation.

We have tried to find examples whereby Bafilomycin A1 treatment prevents the formation of Gal3 punctate. We have failed to find examples so far. Typically, Bafilomycin A1 is used to block the subsequent fusion of damaged lysosomes with healthy lysosomes, ie lysophagy (https://doi.org/10.1073/pnas.2312306120). In the original paper that describes lysophagy after LLOMe treatment, HeLa cells still contained Gal3 puncta 24 hours post treatment with LLOMe and Bafilomycin A1 (early timepoints were not analysed) (<https://doi.org/10.1038/emboj.2013.171>) and the figure below showing GFP-mCherry tagged Gal3 puncta, whereby GFP signal is pH dependent.

Furthermore, LLOMe + BafA1 still caused the formation of Gal3 puncta, but which no longer colocalized with LC3 puncta as shown below, published by (<https://doi.org/10.1083/jcb.202303078>). ­­­

­

The only evidence we could find that Bafilomycin A1 may interfere with LLO-Me activity is derived from papers suggested by reviewer 3. For instance, the paper (<https://doi.org/10.1038/s41586-023-06726-w>) states that “Given that BAFA1 could interfere with the lysosomal processing of LLOMe^27^,…” whereby reference 27 is the Thiele and Lipsky, PNAS, 1990 paper. However, Thiele and Lipsky paper demonstrates that ammonium chloride raised lysosomal pH but did not prevent LLO-Me processing. Despite this, the paper 10.1038/s41467-022-34632-8 shows that Bafilomycin A1 impairs LLOMe-mediated “protease leakage-dependent mitochondrial protein and activity decrease (Supplementary Fig. 3g, h)”.

Unless reviewer 3 and the academic editor point us to published examples showing that Bafilomycin A1 prevents LLO-Me Gal3 puncta formation, we conclude that our results are supported by the literature.

As suggested by reviewer 3, we have now increased the resolution of the image by applying super-resolution microscopy, including multiple cells and zoom-in sections (Figure S12B).

Minor:

Including additional markers such as LAMP-1 and DAPI would improve the panel. Figures do not present a scale bar.

We have now included DAPI and scale bars (Figure S12B).

The authors show lysosome damage and mitochondrial activity impairment phenotypes. This paper should be cited or discussed: 10.1038/s41467-022-34632-8.

We have now cited the above paper to highlight that lysosomal damage can directly affect mitochondrial health.

Of note and not discussed in our manuscript, however, if the above statement that BafA1 does not affect LLOMe mediated lysosomal rupture as determined by Gal3 puncta staining, it is unclear to us how Bafilomycin A1 still prevented mitochondrial membrane protein degradation in LLOMe-treated macrophages. It is possible that the effect of Bafilomycin A1 depends on its concentration, the concentration of LLOMe, cell lines or culture conditions.
